# Characterization of the complete mitochondrial genomes of the zoonotic parasites *Bolbosoma nipponicum* and *Corynosoma villosum* (Acanthocephala: Polymorphida) and the molecular phylogeny of the order Polymorphida

**DOI:** 10.1017/S0031182023001099

**Published:** 2024-01

**Authors:** Dai-Xuan Li, Rui-Jia Yang, Hui-Xia Chen, Tetiana A. Kuzmina, Terry R. Spraker, Liang Li

**Affiliations:** 1 Hebei Key Laboratory of Animal Physiology, Biochemistry and Molecular Biology; Hebei Collaborative Innovation Center for Eco-Environment; College of Life Sciences, Hebei Normal University, 050024 Shijiazhuang, Hebei Province, P. R. China; 2 Hebei Research Center of the Basic Discipline Cell Biology; Ministry of Education Key Laboratory of Molecular and Cellular Biology; 050024 Shijiazhuang, Hebei Province, P. R. China; 3 I. I. Schmalhausen Institute of Zoology National Academy of Sciences of Ukraine, 15, Bogdan Khmelnytsky Street, Kyiv 01054, Ukraine; 4 Institute of Parasitology, Slovak Academy of Sciences, Hlinkova 3, 04001, Košice, Slovak Republic; 5 Diagnostic Laboratory, Department of Microbiology, Immunology and Pathology, College of Veterinary Medicine and Biomedical Sciences, Colorado State University, Fort Collins, CO 80526, USA

**Keywords:** Acanthocephala, *Bolbosoma*, *Corynosoma*, mitochondrial genome, molecular phylogeny, Polymorphidae, zoonotic parasite

## Abstract

Acanthocephalans of the order Polymorphida mainly parasitic in birds and mammals, are of veterinary, medical and economic importance. However, the evolutionary relationships of its 3 families (Centrorhynchidae, Polymorphidae and Plagiorhynchidae) remain under debate. Additionally, some species of Polymorphida (i.e. *Bolbosoma* spp. and *Corynosoma* spp.) are recognized as zoonotic parasites, associated with human acanthocephaliasis, but the mitochondrial genomes for representatives of *Bolbosoma* and *Corynosoma* have not been reported so far. In the present study, the complete mitochondrial genomes *B. nipponicum* and *C. villosum* (Acanthocephala: Polymorphidae) are reported for the first time, which are 14 296 and 14 241 bp in length, respectively, and both contain 36 genes [including 12 PCGs, 22 tRNA genes and 2 rRNA genes] and 2 non-coding regions (*NCR1* and *NCR2*). The gene arrangement of some tRNAs in the mitogenomes of *B. nipponicum* and *C. villosum* differs from that found in all other acanthocephalans, except *Polymorphus minutus*. Phylogenetic results based on concatenated amino acid (AA) sequences of the 12 protein-coding genes (PCGs) strongly supported that the family Polymorphidae is a sister to the Centrorhynchidae rather than the Plagiorhynchidae, and also confirmed the sister relationship of the genera *Bolbosoma* and *Corynosoma* in the Polymorphidae based on the mitogenomic data for the first time. Our present findings further clarified the phylogenetic relationships of the 3 families Plagiorhynchidae, Centrorhynchidae and Polymorphidae, enriched the mitogenome data of the phylum Acanthocephala (especially the order Polymorphida), and provided the resource of genetic data for diagnosing these 2 pathogenic parasites of human acanthocephaliasis.

## Graphical abstract

**Figure:**
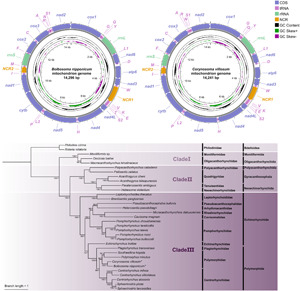

## Introduction

Acanthocephalans (commonly named as spiny- or thorny-headed worms) are an important group of obligate endoparasites occurring in the alimentary track of all major vertebrate groups (Petrochenko and Skrjabin, 1956; Yamaguti, 1963; Nickol, 1985; Naidu, 2012; Amin, 2013), which are of veterinary, medical and economic importance in domestic animals, wildlife and humans (Petrochenko and Skrjabin, 1956; Moore *et al*., 1969; Nickol, 1985). Some acanthocephalan species of the genera *Macracanthorhynchus* Travassos, 1917, *Moniliformis* Travassos, 1915, *Corynosoma* Lühe, 1904, and *Bolbosoma* Porta, 1908, rarely *Pseudoacanthocephalus* Petrochenko, 1956 and *Acanthocephalus* Koelreuther, 1771, are recognized as zoonotic parasites associated with human acanthocephaliasis (Skrinnik *et al*., 1958; Schmidt, 1971; Leng *et al*., 1983; Miyazaki, 1991; Berenji *et al*., 2007; Fujita *et al*., 2016).

The present knowledge regarding the basic molecular phylogenetic framework of the phylum Acanthocephala Rudolphi, 1808 remains far from complete. Previous studies indicated that the mitochondrial genomes play important roles in the phylogenetics, population genetics and species identification of acanthocephalans (Gazi *et al*., 2016; Song *et al*., 2016; Pan and Jiang, 2018; Muhammad *et al*., 2019*a*, 2019*b*, 2020*a*, 2020*b*, 2020*c*; Dai *et al*., 2022; Gao *et al*., 2022). However, the mitogenomes have been sequenced for only 29 acanthocephalan species, which belonged to 13 families in 6 orders. To date, there are approximately 98% of nominal species and 87% of genera in Acanthocephala with their mitogenomes unavailable so far, which somewhat hinder understanding the mitogenomic evolution and phylogenetics in Acanthocephala.

The order Polymorphida Petrochenko, 1956 currently includes 3 families Plagiorhynchidae Golvan, 1960, Centrorhynchidae Van Cleave, 1916 and Polymorphidae Meyer, 1931 (Amin, 2013). Until now, the evolutionary relationships of the 3 families remain under debate. Some previous molecular analyses considered the Plagiorhynchidae and Polymorphidae to have an affinity (García-Varela *et al*., 2013; Gazi *et al*., 2015; Muhammad *et al*., 2020*a*, 2020*b*, 2020*c*); however, other studies supported the Polymorphidae and Centrorhynchidae have closer relationship than the Plagiorhynchidae (Gazi *et al*., 2016; Song *et al*., 2019; Muhammad *et al*., 2019*a*, 2019*b*; Dai *et al*., 2022; Gao *et al*., 2022; Zhao *et al*., 2023).

In Polymorphida, the Polymorphidae is the largest family and contains 12 genera, namely *Andracantha* Schmidt, 1975, *Ardeirhynchus* Dimitrova and Georgiev, 1994, *Arhythmorhynchus* Lühe, 1911, *Bolbosoma*, *Corynosoma*, *Diplospinifer* Fukui, 1929, *Filicollis* Lühe, 1911, *Ibirhynchus* García-Varela, de León, Aznar et Nadler, 2011, *Polymorphus* Lühe, 1911, *Profilicollis* Meyer, 1931, *Pseudocorynosoma* Aznar, de León et Raga, 2006, and *Southwellina* Witenberg, 1932, with over 120 species reported from marine mammals, fish-eating marine birds and waterfowls worldwide (Deliamure, 1968; Schmidt, 1975; Dimitrova and Georgiev, 1994; Aznar *et al*., 2006; García-Varela *et al*., 2011, 2013; Amin, 2013). Several species of *Bolbosoma* and *Corynosoma*, for example, *Bolbosoma* cf. *capitatum, Bolbosoma* sp., *Corynosoma villosum* Van Cleave, 1953, *C. strumosum* (Rudolphi, 1802), *C. validum* Van Cleave, 1953 and *Corynosoma* sp. are recognized as parasites associated with human acanthocephaliasis (Beaver *et al*., 1983; Tada *et al*., 1983; Ishikura *et al*., 1996; Mori *et al*., 1998; Hino *et al*., 2002; Isoda *et al*., 2006; Arizono *et al*., 2012; Fujita *et al*., 2016; Takahashi *et al*., 2016; Kaito *et al*., 2019; Santoro *et al*., 2021). Despite their significance, the mitochondrial genomes for representatives of *Bolbosoma* and *Corynosoma* have not been reported.

In the present study, the complete mitochondrial genomes of *Bolbosoma nipponicum* Yamaguti, 1939 and *C. villosum* (Acanthocephala: Polymorphidae) are sequenced and annotated for the first time, based on specimens collected from the northern fur seal *Callorhinus ursinus* (Linnaeus) (Mammalia: Carnivora) and the Pacific halibut *Hippoglossus stenolepis* (Schmidt) (Pleuronectiformes: Pleuronectidae) in Alaska, USA, which also represented the first mitogenome from the genera *Bolbosoma* and *Corynosoma* (Polymorphida: Echinorhynchidae). Additionally, in order to further clarify the evolutionary relationships of the 3 families Plagiorhynchidae, Centrorhynchidae and Polymorphidae in the order Polymorphida, phylogenetic analyses based on concatenated amino acid (AA) sequences of the 12 protein-coding genes (PCGs) of all available acanthocephalan mitogenomes were performed using Bayesian inference (BI) and maximum likelihood (ML), respectively.

## Materials and methods

### Species identification

The acanthocephalan specimens of *B. nipponicum* and *C. villosum* were collected from the intestine of subadult northern fur seals *Callorhinus ursinus* (Linnaeus) (Mammalia: Carnivora) and *Hippoglossus stenolepis* (Schmidt) (Pleuronectiformes: Pleuronectidae) in St. Paul Island, Alaska, USA, fixed and stored in 70% ethanol. The specimens were identified as *B. nipponicum* and *C. villosum* based on morphological features according to previous studies (Van Cleave, 1953; Margolis, 1956; Ru *et al*., 2022). Voucher specimens were deposited in the College of Life Sciences, Hebei Normal University, Hebei Province, China (*B. nipponicum*: HBNU–A-2022M002L; *C. villosum*: HBNU–A-2022F003L).

### Molecular procedures

The total genomic DNA of each individual of *B. nipponicum* and *C. villosum* was extracted using the Magnetic Universal Genomic DNA Kit (DP705) (Tiangen Biotech, Beijing, China) according to the manufacturer's instructions: (1) cut the sample tissue into small pieces, add 300 *μ*L tissue digestives GHA and 20 *μ*L Proteinase K, and grind the tissue thoroughly; (2) transfer the above-treated sample solution of 300 *μ*L into a new 1.5 mL centrifuge tube; (3) add 300 *μ*L lysate GHL and 300 *μ*L isopropyl alcohol, shake and mix well; (4) add 15 *μ*L magnetic bead suspension GH, shake and mix for 1 min, stand for 9 min in total, shake and mix for 1 min each 3 mins; (5) the centrifuge tube was placed on the magnetic rack and stood for 30 s. After the magnetic bead was completely absorbed, the liquid was carefully absorbed, and the DNA solution was transferred to the new centrifuge tube. The genomic DNA sample was fragmented by sonication to a size of 350 bp in preparation for genomic library.

A total of 20 GB of gene library data for each sample of *B. nipponicum* and *C. villosum* were yielded using the Pair-End 150 sequencing method on the Illumina NovaSeq 6000 platform by Novogene (Tianjin, China). Program GetOrganelle v1.7.2a (Jin *et al*., 2020) was employed to reconstruct the mitochondrial genomes of these acanthocephalans. The locations of PCGs, transfer RNA (tRNA), and ribosomal RNA (rRNA) in the generated mitochondrial genomes were roughly annotated using MitoZ v2.4 (Meng *et al*., 2019) and web-servers MITOS (http://mitos.bioinf.uni-leipzig.de/index.py). All PCGs were confirmed manually using the ORF finder (https://www.ncbi.nlm.nih.gov/orffinder/) based on the invertebrate mitochondrial genetic code. Transfer RNA genes were additionally identified using BLAST-based on a database of the existing tRNA sequences of Acanthocephala. The secondary structures of tRNAs were predicted by the ViennaRNA module (Gruber *et al*., 2015) and then manually corrected building on MitoS2 (Bernt *et al*., 2007) and RNAstructure v6.3 (Reuter and Mathews, 2010). The CGView online server V1.0 (http://stothard.afns.ualberta.ca/cgview_server/) was used to generate the circular genomic maps. The base composition, amino acid usage and relative synonymous codon usage (RSCU) were calculated by Python script (details see the Supplementary material), which refers to codon adaptation index (Cock *et al*., 2009). Strand asymmetry was calculated using the formulae: AT-skew = (A − T)/(A + T); GC-skew = (G − C)/(G + C). The complete mitochondrial genomes of *B. nipponicum* and *C. villosum* obtained herein were deposited in the GenBank database (http://www.ncbi.nlm.nih.gov, under the accession numbers *Corynosoma villosum*: OR468095, *Bolbosoma nipponicum*: OR468096).

### Phylogenetic analyses

Phylogenetic analyses were conducted based on concatenated amino acid (AA) sequences of the 12 PCGs using BI and ML, respectively. Two species of Bdelloidea, *Rotaria rotatoria* (NC013568.1) and *Philodina citrina* (FR856884.1) were chosen as the out-group. The in-group included the newly sequenced *B. nipponicum* and *C. villosum* and the other 29 species of acanthocephalans with mitogenomic data. Detailed information on representatives included in the present phylogeny was provided in Table 1. For phylogenetic analyses, PhyloSuite was used to collect all mitogenomic sequences from GenBank files, standardize annotation and extract mitogenomic data (Zhang *et al*., 2020). The extracted amino acid sequences of all 12 PCGs were aligned in MAFFT v7.313 under iterative refinement method of E-INS-I (Katoh and Standley, 2013). The AAs dataset was concatenated into a single alignment by PhyloSuite, respectively. Substitution models were compared and selected according to the Bayesian Information Criterion (BIC) by using ModelFinder (Kalyaanamoorthy *et al*., 2017). The VT + F + I + G4 was identified as the optimal amino acid substitution model for both partitions (partition1: *cox1*, *cox2*, *nad1*; partition2: all other genes). Bayesian Information Criterion analysis settings were lser nst = 6, rates = invgamma, mcmc ngen = 5 000 000, printfreq = 1000, samplefreg = 100, nchains = 4, nruns = 2. The analysis continued until the average standard deviation of split frequencies was lower than 0.01. The first 25% of trees were treated as ‘burn-in’. For ML analysis, 1000 bootstrap replicates were used to calculate the bootstrap of the program in IQTREE v2.1.2, keep the default values for other parameters (Golombek *et al*., 2015; Minh *et al*., 2020). The iTOL v6.1.1 was used to visualize the phylogeny and architecture using files generated by PhyloSuite (Letunic and Bork, 2021).

**Table 1.** Detailed information of representatives with their mitogenomic data included in the present phylogeny

Phylum/Class	Order	Family	Species	Accession	Length	AT%	References
Out-group							
Rotifera							
Eurotatoria	Bdelloidea	Philodinidae	*Rotaria rotatoria*	NC_013568	15 319	73.2	(Min and Park, 2009)
			*Philodina citrina*	FR856884	14 003	77.7	(Weber *et al*., 2013)
In-group							
Acanthocephala							
Archiacanthocephala	Moniliformida	Moniliformidae	*Moniliformis* sp.	OK415026	14 066	66.2	(Dai *et al*., 2022)
	Oligacanthorhynchida	Oligacanthorhynchidae	*Macracanthorhynchus hirudinaceus*	NC_019808	14 282	65.2	(Weber *et al*., 2013)
			*Oncicola luehei*	NC_016754	14 281	60.2	(Gazi *et al*., 2012)
Eoacanthocephala	Gyracanthocephala	Quadrigyridae	*Acanthogyrus bilaspurensis*	MT476589	13 360	59.3	unpublished
			*Acanthogyrus cheni*	KX108947	13 695	65.3	(Song *et al*., 2016)
			*Pallisentis celatus*	NC_022921	13 855	61.5	(Pan and Nie, 2013)
	Neoechinorhynchida	Neoechinorhynchidae	*Hebesoma violentum*	KC415004	13 393	59.4	(Pan and Jiang, 2018)
		Tenuisentidae	*Paratenuisentis ambiguus*	NC_019807	13 574	66.9	(Weber *et al*., 2013)
Palaeacanthocephala	Echinorhynchida	Arhythmacanthidae	*Heterosentis pseudobagri*	OP278658	13 742	62.5	(Gao *et al*., 2023)
		Cavisomidae	*Cavisoma magnum*	MN562586	13 594	63.0	(Muhammad *et al*., 2020*a*)
		Echinorhynchidae	*Echinorhynchus truttae*	NC_019805	13 659	63.1	(Weber *et al*., 2013)
		Leptorhynchoidiae	*Brentisentis yangtzensis*	MK651258	13 864	68.3	(Song *et al*., 2019)
			*Leptorhynchoides thecatus*	NC_006892	13 888	71.4	(Steinauer *et al*., 2005)
		Pomphorhynchidae	*Pomphorhynchus bulbocolli*	JQ824371	13 915	59.9	unpublished
			*Pomphorhynchus laevis*	JQ809446	13 889	57.1	unpublished
			*Pomphorhynchus rocci*	JQ824373	13 845	60.7	unpublished
			*Pomphorhynchus tereticollis*	JQ809451	13 965	56.9	unpublished
			*Pomphorhynchus zhoushanensis*	MN602447	14 565	56.0	unpublished
		Pseudoacanthocephalidae	*Pseudoacanthocephalus bufonis*	MZ958236	14 056	58.4	(Zhao *et al*., 2023)
		Rhadinorhynchidae	*Micracanthorhynchina dakusuiensis*	OP131911	16 309	56.8	(Gao *et al*., 2022)
	Polymorphida	Centrorhynchidae	*Centrorhynchus clitorideus*	MT113355	15 884	55.5	(Muhammad *et al*., 2020*c*)
			*Centrorhynchus milvus*	MK922344	14 314	54.5	(Muhammad *et al*., 2019*b*)
			*Centrorhynchus aluconis*	KT592357	15 144	54.5	(Gazi *et al*., 2016)
			*Sphaerirostris lanceoides*	MT476588	13 478	58.0	(Muhammad *et al*., 2020*b*)
			*Sphaerirostris picae*	MK471355	15 170	58.1	(Muhammad *et al*., 2019*a*)
		Polymorphidae	*Polymorphus minutus*	MN646175	14 149	64.4	(Sarwar *et al*., 2021)
			*Southwellina hispida*	NC_026516	14 742	63.9	(Gazi *et al*., 2015)
			***Bolbosoma nipponicum***		**14 296**	**60**.**9**	**present study**
			***Corynosoma villosum***		**14 241**	**61**.**0**	**present study**
		Plagiorhynchidae	*Plagiorhynchus transversus*	NC_029767	15 477	61.1	(Gazi *et al*., 2016)
Polyacanthocephala	Polyacanthorhynchida	Polyacanthorhynchidae	*Polyacanthorhynchus caballeroi*	NC_029766	13 956	56.3	(Gazi *et al*., 2016

*Bolbosoma nipponicum* and *Corynosoma villosum* in the present study are indicated in bold.

## Results

### General characterization of the complete mitogenomes of *B. nipponicum* and *C. villosum*

#### Gene content and organization of mitogenomes

**Figure 1.**
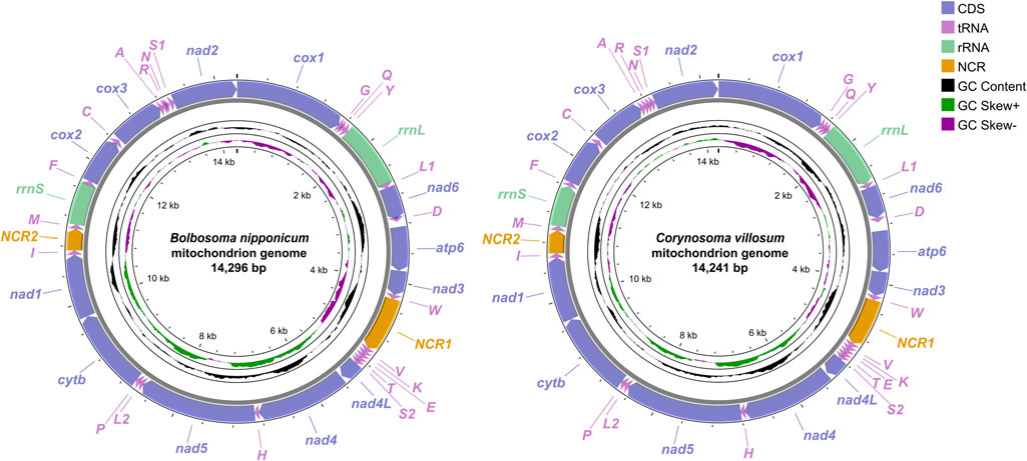
Gene maps of the mitochondrial genomes of *Bolbosoma nipponicum* and *Corynosoma villosum*. All genes are transcribed in the clockwise direction on the same strand, and 22 tRNA genes are designated by the 1-letter code with numbers differentiating each of the 2 tRNAs serine and leucine. The outermost circle shows the GC content and the innermost circle shows the GC skew.

The complete mitochondrial genomes of *B. nipponicum* and *C. villosum* are 14 296 and 14 241 bp in length, respectively, and both contain 36 genes, including 12 PCGs (missing *atp8*) (*cox1–3*, *cytb*, *nad1–6*, *nad4L* and *atp6*), 22 tRNA genes and 2 rRNA genes (*rrnL* and *rrnS*) (Fig. 1, Table 2). All genes are transcribed from the same strand. Two non-coding regions (*NCR1* and *NCR2*), are present in the same positions in the mitogenomes of *B. nipponicum* and *C. villosum* (*NCR1* is 776 bp in *B. nipponicum vs* 676 bp in *C. villosum*, both located between *trnW* and *trnV*; *NCR2* is 301 bp in *B. nipponicum vs* 318 bp in *C. villosum*, both between *trnI* and *trnM*) (Fig. 1; Table 2). The overall A + T contents in the mitogenomes of *B. nipponicum* and *C. villosum* are 60.88% and 60.99%, respectively (Table 3), both displaying strong A + T bias.

**Table 2.** Organization of the mitochondrial genomes of *Bolbosoma nipponicum* and *Corynosoma villosum*

*Bolbosoma nipponicum*						*Corynosoma villosum*					
Gene/Region	Position 5′ to 3′	Size (bp)	Ini/Ter cod	Anticodon	Int seq	Gene/Region	Position 5′ to 3′	Size (bp)	Ini/Ter cod	Anticodon	Int seq
*cox1*	1–1542	1542	TTG/TAG		-2	*cox1*	1–1536	1536	GTG/TAA		0
*trnG*	1541–1593	53		uuc	-12	*trnG*	1537–1591	55		ucc	-5
*trnQ*	1582–1631	50		uua	7	*trnQ*	1587–1647	61		uug	-13
*trnY*	1639–1691	53		gua	0	*trnY*	1635–1687	53		gua	0
*rrnL*	1692–2604	913			0	*rrnL*	1688–2601	914			0
*trnL1*	2605–2657	53		uag	0	*trnL1*	2602–2653	52		uag	0
*nad6*	2658–3119	462	GTG/TAG		-23	*nad6*	2654–3095	442	GTG/T		0
*trnD*	3097–3149	53		guc	82	*trnD*	3096–3149	54		guc	0
*atp6*	3232–3840	609	ATT/TAG		-1	*atp6*	3279–3842	564	ATG/TAA		129
*nad3*	3840–4185	346	GTG/T		0	*nad3*	3842–4187	346	ATG/T		0
*trnW*	4186–4246	61		uca	0	*trnW*	4188–4248	61		uca	0
*NCR1*	4247–5022	776			0	*NCR1*	4249–4924	676			0
*trnV*	5023–5081	59		uac	0	*trnV*	4925–4985	61		uac	0
*trnK*	5082–5134	53		uuu	1	*trnK*	4986–5040	55		uuu	3
*trnE*	5136–5187	52		uuc	2	*trnE*	5044–5095	52		uuc	0
*trnT*	5190–5245	56		ugu	-7	*trnT*	5096–5151	56		ugu	-4
*trnS2*	5239–5291	53		uga	-1	*trnS2*	5148–5201	54		uga	-1
*nad4L*	5291–5560	270	GTG/TAA		14	*nad4L*	5201–5470	270	GTG/TAA		14
*nad4*	5575–6847	1273	ATG/T		0	*nad4*	5485–6757	1273	ATG/T		0
*trnH*	6848–6902	55		gug	0	*trnH*	6758–6809	52		gug	0
*nad5*	6903–8549	1647	GTG/TAG		0	*nad5*	6810–8456	1647	GTG/TAG		0
*trnL2*	8550–8603	54		uaa	2	*trnL2*	8457–8510	54		uaa	5
*trnP*	8606–8658	53		ugg	0	*trnP*	8516–8569	54		ugg	0
*cytb*	8659–9795	1137	GTG/TAA		-1	*cytb*	8570–9706	1137	GTG/TAG		0
*nad1*	9795–10 685	891	ATG/TAG		1	*nad1*	9707–10 600	894	GTG/TAG		2
*trnI*	10 687–10 743	57		gau	0	*trnI*	10 603–10 657	55		gau	0
*NCR2*	10 744–11 044	301			0	*NCR2*	10 658–10 975	318			0
*trnM*	11 045–11 098	54		cau	0	*trnM*	10 976–11 031	56		cau	0
*rrnS*	11 099–11 683	585			0	*rrnS*	11 032–11 615	584			0
*trnF*	11 684–11 737	54		gaa	0	*trnF*	11 616–11 675	60		gaa	-6
*cox2*	11 738–12 391	654	GTG/TAA		-2	*cox2*	11 670–12 323	654	GTG/TAA		-1
*trnC*	12 390–12 443	54		gca	17	*trnC*	12 323–12 376	54		gca	18
*cox3*	12 461–13 180	720	GTG/TAA		-2	*cox3*	12 395–13 114	720	GTG/TAA		0
*trnA*	13 179–13 231	53		ugc	0	*trnA*	13 115–13 169	55		ugc	0
*trnR*	13 232–13 292	61		acg	-31	*trnR*	13 170–13 229	60		acg	-12
*trnN*	13 262–13 321	60		guu	13	*trnN*	13 218–13 281	64		guu	-9
*trnS1*	13 335–13 390	56		acu	0	*trnS1*	13 273–13 330	58		acu	-1
*nad2*	13 391–14 296	906	GTG/TAG		0	*nad2*	13 330–14 235	906	GTG/TAG		6

Ini/Ter cod: initial/terminal codons, Int seq: intergenic sequences.

*Bolbo*.

**Table 3.** Base composition and skewness in the mitogenomes of *Bolbosoma nipponicum* and *Corynosoma villosum*

Location/Species	A%	T%	C%	G%	AT%	AT-skew	GC-skew	Total
***Bolbosoma nipponicum***								
Whole mitochondrial genome	20.15	40.72	8.86	30.26	60.88	−0.34	0.55	14 296
Protein coding genes (PCGs)	17.72	42.09	8.21	31.99	59.81	−0.41	0.59	10 457
1st codon	21.48	32.95	8.98	36.59	54.43	−0.21	0.61	3487
2nd codon	14.35	49.10	10.82	25.74	63.44	−0.55	0.41	3485
3rd codon	17.33	44.22	4.82	33.63	61.55	−0.44	0.75	3485
tRNAs	25.10	36.87	10.60	27.42	61.97	−0.19	0.44	1207
rRNAs	26.97	35.05	10.68	27.30	62.02	−0.13	0.44	1498
rrnS	28.89	35.04	9.91	26.15	63.93	−0.10	0.45	585
rrnL	25.74	35.05	11.17	28.04	60.79	−0.15	0.43	913
Non-coding region 1	29.90	38.40	11.47	20.23	68.30	−0.12	0.28	776
Non-coding region 2	29.24	40.86	9.30	20.60	70.10	−0.17	0.38	301
***Corynosoma villosum***								
Whole mitochondrial genome	21.24	39.74	7.60	31.41	60.99	−0.30	0.61	14 241
Protein coding genes (PCGs)	18.79	41.09	7.19	32.93	59.88	−0.37	0.64	10 389
1st codon	22.05	32.70	7.30	37.95	54.75	−0.19	0.68	3465
2nd codon	14.70	49.60	9.82	25.88	64.30	−0.54	0.45	3462
3rd codon	19.61	40.99	4.45	34.95	60.60	−0.35	0.77	3462
tRNAs	26.62	37.06	9.06	27.27	63.67	−0.16	0.50	1236
rRNAs	28.57	34.31	10.35	26.77	62.88	−0.09	0.44	1498
rrnS	29.28	33.56	9.76	27.40	62.84	−0.07	0.47	584
rrnL	28.12	34.79	10.72	26.37	62.91	−0.11	0.42	914
Non-coding region 1	28.40	36.98	6.95	27.66	65.38	−0.13	0.60	676
Non-coding region 2	30.82	38.36	5.66	25.16	69.18	−0.11	0.63	318

*Bolbosoma nipponicum* and *Corynosoma villosum* in the present study are indicated in bold.

### Protein-coding genes and codon usage

The 12 PCGs of the mitogenomes of *B. nipponicum* and *C. villosum* are 10 468 bp and 10 389 bp in total length (excluding termination codons), and ranged from 270 bp (*nad4L*) to 1,647 bp (*nad5*) in size (Table 2). Among the 12 PCGs of *B. nipponicum*, 8 genes (*cox2*, *cox3*, *cytb*, *nad2*, *nad3*, *nad4L*, *nad5* and *nad6*) used GTG as the start codon, whereas 2 genes (*nad1* and *nad4*) used ATG. ATT and TTG were used by the *atp6* and *cox1* genes, respectively. TAG was the most commonly used termination codon for 6 genes (*atp6*, *cox1*, *nad1*, *nad2*, *nad5* and *nad6*); 4 genes (*cox2*, *cox3*, *cytb* and *nad4L*) used TAA. The incomplete termination codon T was inferred for the *nad3* and *nad4* genes (Table 2).

**Figure 2.**
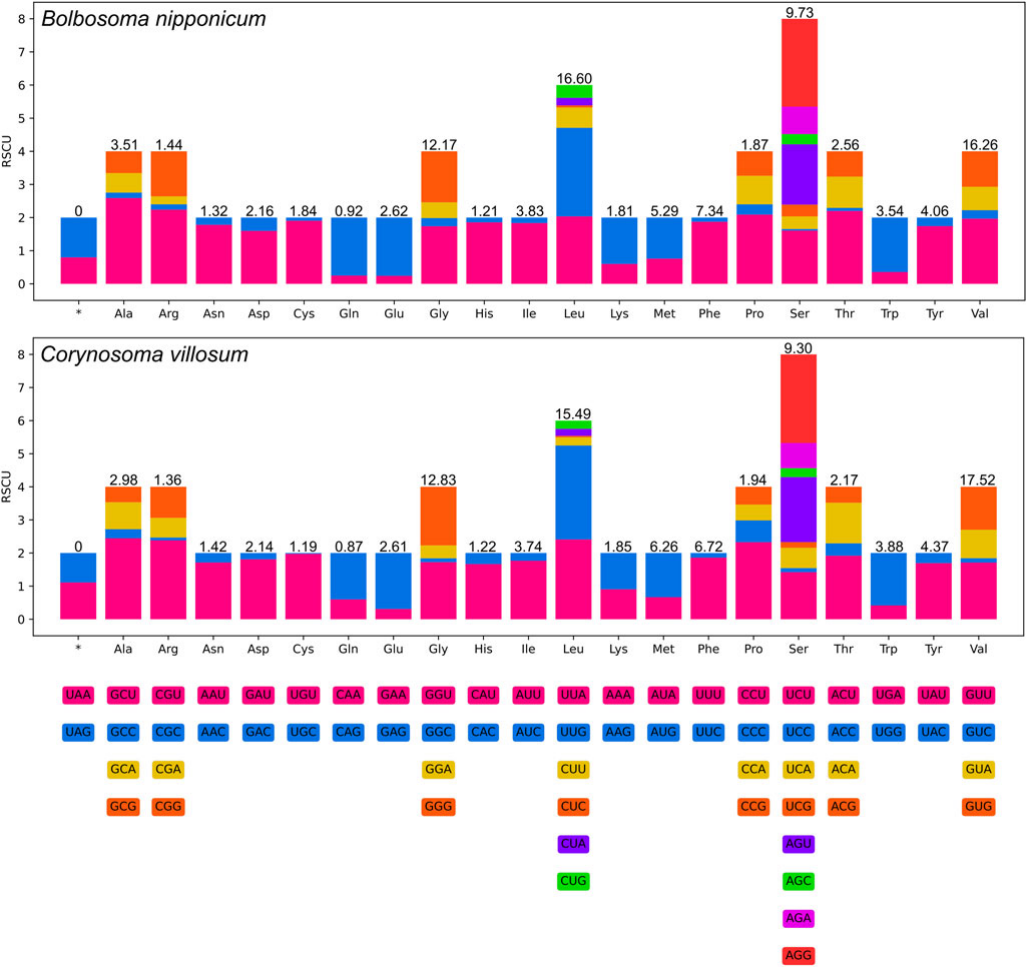
Relative synonymous codon usage (RSCU) of *Bolbosoma nipponicum* and *Corynosoma villosum*. Codon families (in alphabetical order) are provided below the horizontal axis. Values on the top of each bar represent amino acid usage in percentage.

Among the 12 PCGs of *C. villosum*, 9 genes (*cox1*, *cox2*, *cox3*, *cytb*, *nad1*, *nad2*, *nad4L*, *nad5* and *nad6*) used GTG as the start codon, whereas 3 genes (*atp6*, *nad3* and *nad4*) used ATG. TAA was the most commonly used termination codon for 6 genes (*atp6*, *cox1*, *cox2*, *cox3*, *nad2* and *nad4L*); 3 genes (*cytb*, *nad1* and *nad5*) used TAG. The incomplete termination codon T was inferred for the *nad3*, *nad4* and *nad6* genes (Table 2). RSCU is summarized in Fig. 2.

### Transfer and ribosomal RNAs

**Figure 3.**
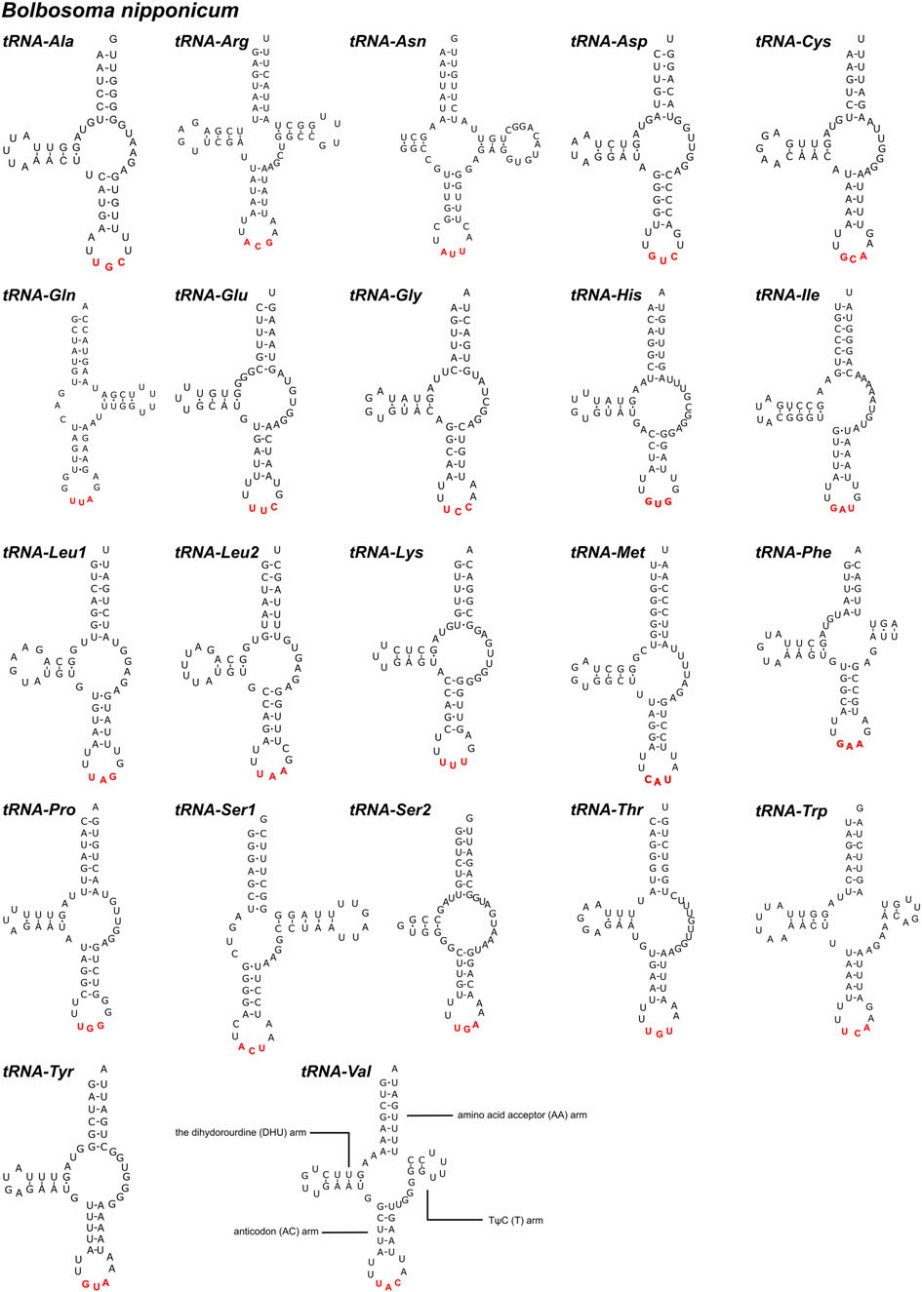
The predicted secondary structures of 22 tRNAs in the mitogenome of *Bolbosoma nipponicum* (Watson–Crick bonds indicated by lines, GU bonds indicated by dots, red bases representing anticodons). The tRNAs are labelled with the abbreviations of their corresponding amino acids according to the IUPAC-IUB code.

**Figure 4.**
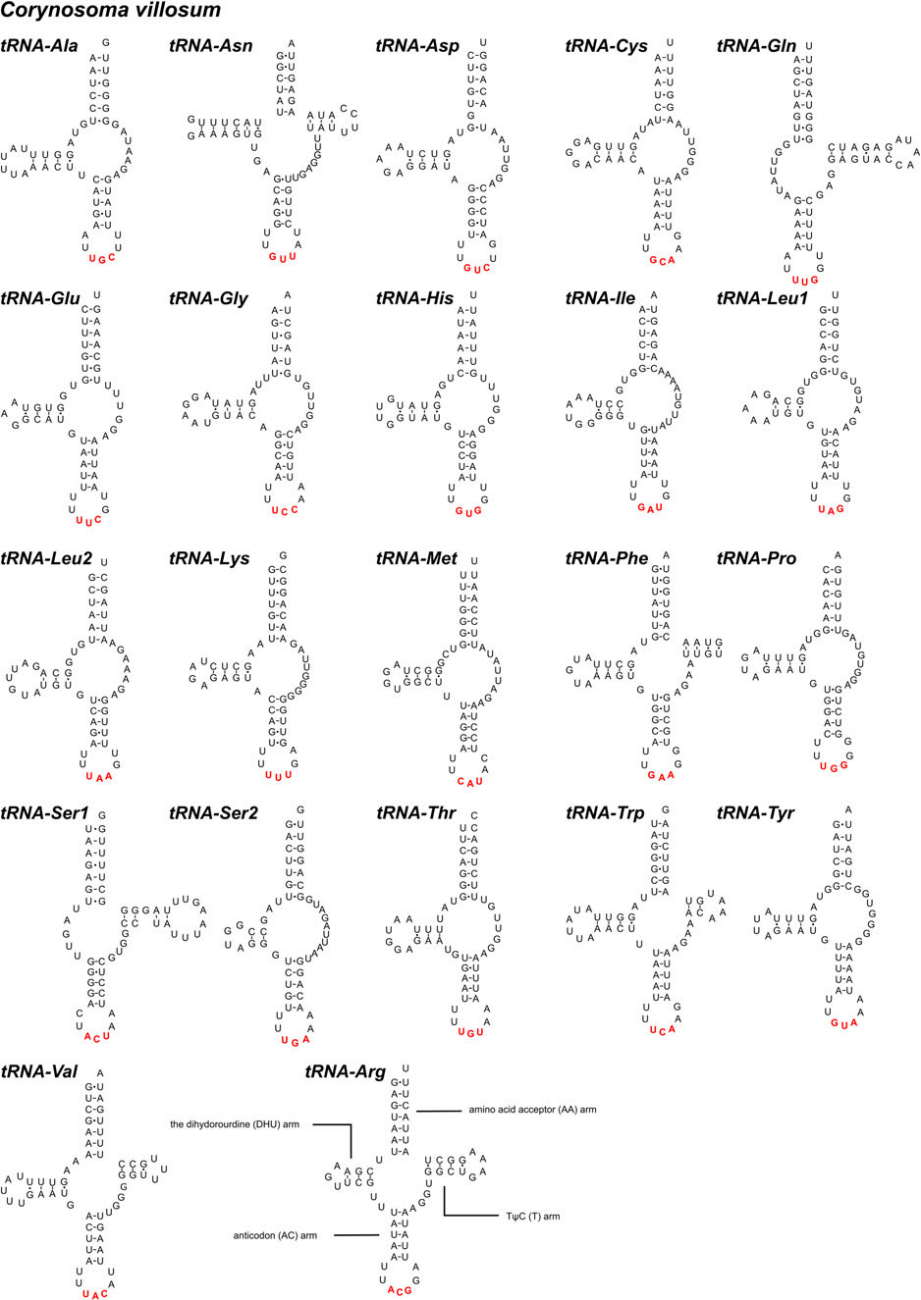
The predicted secondary structures of 22 tRNAs in the mitogenome of *Corynosoma villosum* (Watson–Crick bonds indicated by lines, GU bonds indicated by dots, red bases representing anticodons). The tRNAs are labelled with the abbreviations of their corresponding amino acids according to the IUPAC-IUB code.

In the mitogenomes of *B. nipponicum* and *C. villosum*, 22 tRNAs are identified with lengths ranging from 50 to 61 bp in *B. nipponicum*, and from 52 to 64 bp in *C. villosum* (Fig. 1, Table 2). Their anticodon secondary structures are provided (Figs 3, 4). In the 22 tRNAs of *B. nipponicum* and *C. villosum*, 5 tRNAs (*trnR*, *trnN*, *trnF*, *trnW* and *trnV*) were predicted to be folded into typical cloverleaf secondary structure, 2 tRNAs (*trnQ* and *trnS1*) lacked dihydorourdine (DHU) arm, the remaining 15 tRNAs lacked TΨC arm (Figs 3, 4, Table 2).

Two rRNAs (*rrnL* and *rrnS*) were identified in the mitogenomes of *B. nipponicum* and *C. villosum* (*rrnL* is 913 bp in *B. nipponicum vs* 914 bp in *C. villosum*, both located between *trnY* and *trnL1*; *rrnS* is 585 bp in *B. nipponicum vs* 584 bp in *C. villosum*, both located between *trnM* and *trnF*) (Fig. 1, Table 2).

### Gene order

**Figure 5.**
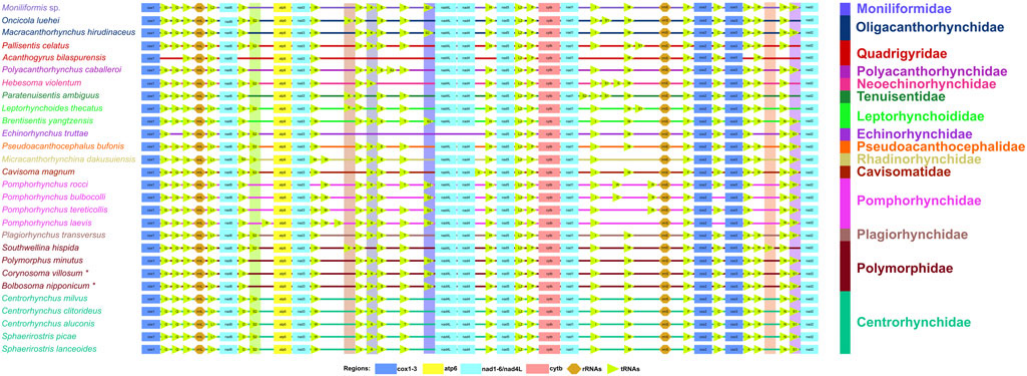
Comparison of the linearized mitochondrial genome arrangement for acanthocephalans species. All genes are transcribed in the same direction from left to right. The tRNAs are labelled by single-letter code for the corresponding amino acid. *Bolbosoma nipponicum* and *Corynosoma villosum* are indicated using asterisk (*).

The arrangements of the 36 genes in the mitogenomes of *B. nipponicum* and *C. villosum* are identical, both in the following order: *cox1, trnG, trnQ, trnY, rrnL, trnL1, nad6, trnD, atp6, nad3, trnW, trnV, trnK, trnE, trnT, trnS2, nad4L, nad4, trnH, nad5, trnL2, trnP, cytb, nad1, trnI, trnM, rrnS, trnF, cox2, trnC, cox3, trnA, trnR, trnN, trnS1, nad2* (Fig. 5).

### Phylogenetic analyses

**Figure 6.**
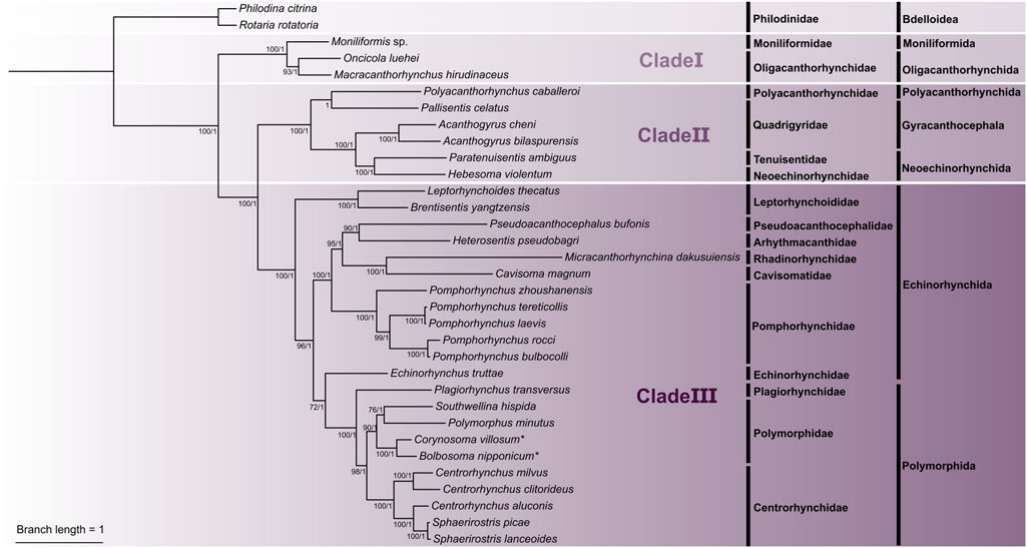
Phylogenetic analyses of Acanthocephala inferred from ML and BI methods based on concatenated amino acid sequences of 12 PCGs of mitochondrial genomes. *Rotaria rotatoria* and *Philodina citrina* were chosen as the out-group. Bootstrap values ⩾70 and Bayesian posterior probabilities values ⩾0.70 are shown in the phylogenetic trees. *Bolbosoma nipponicum* and *Corynosoma villosum* are indicated using asterisk (*).

In the present study, phylogenetic trees based on concatenated amino acid sequences of the 12 PCGs using ML and BI methods have nearly same topologies, except the supported value of some branches, which all showed that the representatives of the phylum Acanthocephala were divided into 3 large monophyletic clades (Figs 6). Clade I includes *Moniliformis* sp., *Macracanthorhynchus hirudinaceus* and *Oncicola luehei*, which represents the class Archiacanthocephala at the most basal position of the phylogenetic trees. Clade II contains the representatives of the class Eoacanthocephala (*Acanthogyrus bilaspurensis*, *A. cheni*, *Hebesoma violentum*, *Paratenuisentis ambiguus* and *Pallisentis celatus*) and *Polyacanthorhynchus caballeroi* (belonging to the class Polyacanthocephala). Clade III is composed of the representatives of the class Palaeacanthocephala, of which the order Echinorhynchida (including *Cavisoma magnum*, *Echinorhynchus truttae*, *Pseudoacanthocephalus bufonis*, *Brentisentis yangtzensis*, *Pomphorhynchus* spp., *Leptorhynchoides thecatus* and *Micracanthorhynchina dakusuiensis*) is a non-monophyletic group, but the order Polymorphida (including *Plagiorhynchus transversus*, *Polymorphus minutus*, *Southwellina hispida*, *Centrorhynchus* spp., *Sphaerirostris* spp., *Bolbosoma nipponicum* and *Corynosoma villosum*) is a monophyletic group. In the order Polymorphida, the family Polymorphidae is a sister to the family Centrorhynchidae. *Bolbosoma nipponicum* and *Corynosoma villosum* clustered together with strong nodal support (BPP = 1, ML-BP = 100) in all BI and ML trees.

## Discussion

The order Polymorphida is a large group of Acanthocephala, containing over 370 nominal species mainly parasitic in birds and mammals, rarely in reptiles (Amin, 2013; Zhao *et al*., 2020; Ru *et al*., 2022). According to the traditional classification, Polymorphida was divided into 3 families (Centrorhynchidae, Polymorphidae, and Plagiorhynchidae), including 24 genera (Amin, 2013). However, only 8 species representing 5 genera with their mitochondrial genomes have been documented (Gazi *et al*., 2015, 2016; Muhammad *et al*., 2019*a*, 2019*b*, 2020*a*, 2020*b*; Sarwar *et al*., 2021). In Polymorphida, the size of mitochondrial genomes of *B. nipponicum* and *C. villosum* is similar to that of the polymorphid species *Polymorphus minutus* and *Southwellina hispida*; however, the overall A + T contents in the mitogenomes of *B. nipponicum* and *C. villosum* are slightly lower than that of *P. minutus* and S. *hispida* (60.88–60.99% in *B. nipponicum* and *C. villosum vs* 63.9–64.4% in the latter 2 species) (Gazi *et al*., 2015; Sarwar *et al*., 2021), but distinctly higher than that of centrorhynchid species (54.5–58.1%) (Muhammad *et al*., 2019*a*, 2019*b*, 2020*a*, 2020*b*; Gazi *et al*., 2016).

Previous studies indicated that the arrangements of the 12 PCGs and 2 rRNAs in the phylum Acanthocephala are highly conserved, while the position of tRNAs usually varied among different families, genera or species (Gazi *et al*., 2012, 2016; Dai *et al*., 2022; Gao *et al*., 2022; Zhao *et al*., 2023). In the family Polymorphidae, the arrangements of the 36 genes in the mitogenomes of *B. nipponicum* and *C. villosum* are identical to that of *P. minutus* (Sarwar *et al*., 2021), but the positions of some tRNAs are different from that of the polymorphid species *S. hispida* (Gazi *et al*., 2015) and all of the species of Centrorhynchidae and Plagiorhynchidae (Muhammad *et al*., 2019*a*, 2019*b*, 2020*a*, 2020*b*; Gazi *et al*., 2016).

Recent mitogenomic phylogenies brought substantial changes to the traditional classification of Acanthocepha. However, phylogenetic relationships within many lineages of the class Palaeacanthocephala remain insufficiently resolved, due to large numbers of taxa (i.e. the order Heteramorphida, and the families of llliosentidae, Isthmosacanthidae, Heteracanthocephalidae, Fessisentidae, Diplosentidae, Transvenidae, Spinulacorpidae Hypoechinorhynchidae and Gymnorhadinorhynchidae of the order Echinorhynchida) that have not been included yet. Although all of the 3 family-level taxa (Plagiorhynchidae, Centrorhynchidae and Polymorphidae) of the order Polymorphida have been included in some previous mitogenomic phylogenetic studies, very limited genus/species-level taxa have been covered in each family. The evolutionary relationships of the 3 families in Polymorphida and its included genera of each family remain unsolved.

The present mitogenomic phylogenies showed that the order Polymorphida is a monophyletic group, but rejected the monophyly of the order Echinorhynchida, in agreement with the previous studies (García-Varela and Nadler, 2006; García-Varela and de León, 2008; Verweyen *et al*., 2011; García-Varela *et al*., 2013; Braicovich *et al*., 2014). The previous molecular phylogenetic results using single or several concatenated genetic markers (i.e. 18S, 18S + 28S + *cox*1) (Near *et al*., 1998; García-Varela *et al*., 2013) and some recent mitogenomic phylogenies (Muhammad *et al*., 2020*a*, 2020*b*, 2020*c*; Sarwar *et al*., 2021) indicated that the Plagiorhynchidae is a sister to the Polymorphidae or Centrorhynchidae. However, the present phylogenetic results strongly displayed that the Polymorphidae and Centrorhynchidae are more closely related to each other than to the Plagiorhynchidae (Gazi *et al*., 2016; Muhammad *et al*., 2019*a*, 2019*b*; Song *et al*., 2019; Dai *et al*., 2022; Gao *et al*., 2022; Zhao *et al*., 2023). Although Zhao *et al*.’ (2023) study also suggests a close affinity between the Polymorphidae and Centrorhynchidae, there are only 2 representatives of the Polymorphidae in their phylogeny, and the supported value for the close phylogenetic relationship of the Polymorphidae and Centrorhynchidae is weak. However, the present phylogenetic study including 2 additional genus-level taxa of the Polymorphidae, showed strong support for the close affinity between the Polymorphidae and Centrorhynchidae in both ML (BS = 98) and BI (BPP = 1).

Our phylogenetic results also revealed the genus *Bolbosoma* has a sister relationship with *Corynosoma* based on the mitogenomic data for the first time, in concordance with previous studies based on single or several concatenated genetic markers (García-Varela *et al*., 2013; Presswell *et al*., 2018; Ru *et al*., 2022). Moreover, the present findings further clarified the phylogenetic relationships of the 3 families Plagiorhynchidae, Centrorhynchidae and Polymorphidae, enriched the mitogenome data of the phylum Acanthocephala (especially the order Polymorphida), and also provided the resource of genetic data for diagnosing these 2 pathogenic parasites of human acanthocephaliasis.

## Supporting information

Li et al. supplementary material Li et al. supplementary material

## Data Availability

The complete mitochondrial genomes of *B. nipponicum* and *C. villosum* obtained herein were deposited in the GenBank database (http://www.ncbi.nlm.nih.gov, under the accession numbers *Corynosoma villosum*: OR468095, *Bolbosoma nipponicum*: OR468096). Voucher specimens of *B. nipponicum* and *C. villosum* were deposited in the College of Life Sciences, Hebei Normal University, Hebei Province, China.

## Supplementary material

The supplementary material for this article can be found at https://doi.org/10.1017/S0031182023001099
