# Supplementary material for: Characterization of the complete mitochondrial genomes of the zoonotic parasites Bolbosoma nipponicum and Corynosoma villosum (Acanthocephala: Polymorphida) and the molecular phylogeny of the order Polymorphida
Source: Parasitology. 2023 Nov 13;151(1):45–57. doi: 10.1017/S0031182023001099 (PMC10941042; doi:10.1017/S0031182023001099)
Supplement: Li et al. supplementary material [file S0031182023001099sup001.docx]

From collections import Counter

import matplotlib.pyplot as plt

from pandas import DataFrame

from Bio import SeqIO

import pandas as pd

import numpy as np

import argparse

table5 = { "TTT": "Phe", "TTC": "Phe", "TTA": "Leu", "TTG": "Leu",

"TCT": "Ser", "TCC": "Ser", "TCA": "Ser", "TCG": "Ser",

"TAT": "Tyr", "TAC": "Tyr", "TAA": " * ", "TAG": " * ",

"TGT": "Cys", "TGC": "Cys", "TGA": "Trp", "TGG": "Trp",

"CTT": "Leu", "CTC": "Leu", "CTA": "Leu", "CTG": "Leu",

"CCT": "Pro", "CCC": "Pro", "CCA": "Pro", "CCG": "Pro",

"CAT": "His", "CAC": "His", "CAA": "Gln", "CAG": "Gln",

"CGT": "Arg", "CGC": "Arg", "CGA": "Arg", "CGG": "Arg",

"ATT": "Ile", "ATC": "Ile", "ATA": "Met", "ATG": "Met",

"ACT": "Thr", "ACC": "Thr", "ACA": "Thr", "ACG": "Thr",

"AAT": "Asn", "AAC": "Asn", "AAA": "Lys", "AAG": "Lys",

"AGT": "Ser", "AGC": "Ser", "AGA": "Ser", "AGG": "Ser",

"GTT": "Val", "GTC": "Val", "GTA": "Val", "GTG": "Val",

"GCT": "Ala", "GCC": "Ala", "GCA": "Ala", "GCG": "Ala",

"GAT": "Asp", "GAC": "Asp", "GAA": "Glu", "GAG": "Glu",

"GGT": "Gly", "GGC": "Gly", "GGA": "Gly", "GGG": "Gly", }

##RSCU_count

def get_synonymous_codons(genetic_code_dict):

# invert the genetic code dictionary to map each amino acid to its codons

codons_for_amino_acid = {}

for codon, amino_acid in list(genetic_code_dict.items()):

codons_for_amino_acid[amino_acid] = codons_for_amino_acid.get(amino_acid, [])

codons_for_amino_acid[amino_acid].append(codon)

#print(genetic_code_dict.items())

# create dictionary of synonymous codons

# Example: {'CTT': ['CTT', 'CTG', 'CTA', 'CTC', 'TTA', 'TTG'], 'ATG': ['ATG']...}

return {codon: codons_for_amino_acid[genetic_code_dict[codon]] for codon in list(genetic_code_dict.keys())}

def RSCU(sequences, genetic_code_dict=table5):

if not isinstance(sequences, (list, tuple)):

raise ValueError("Be sure to pass a list of sequences, not a single sequence. To find the RSCU of a single sequence, pass it as a one element list.")

# ensure all input sequences are divisible by three

for sequence in sequences:

if len(sequence) % 3 != 0:

raise ValueError("Input sequence not divisible by three")

if not sequence:

raise ValueError("Input sequence cannot be empty")

# count the number of each codon in the sequences

#sequences =['ACTGTCGTA','TTAATGCTGAGT']

sequences = [[sequence[i:i + 3].upper() for i in range(0, len(sequence), 3)] for sequence in sequences]

# flat list of all codons (to be used for counting)

codons = []

for x in sequences:

codons.extend(x)

# The number of occurrences of each codon was calculated

counts = Counter(codons)

# "if a certain codon is never used in the reference set... assign [its

# count] a value of 0.5" (page 1285)

for codon in table5:

if counts[codon] == 0:

counts[codon] = 0.5

# determine the synonymous codons for the genetic code

synonymous_codons = get_synonymous_codons(genetic_code_dict)

# hold the result as it is being calulated

result = {}

# calculate RSCU values

for codon in genetic_code_dict:

result[codon] = counts[codon] / ((len(synonymous_codons[codon]) ** -1) * (sum((counts[_codon] for _codon in synonymous_codons[codon]))))

#print(counts[codon])

#print(len(synonymous_codons[codon]))

#print(sum((counts[_codon] for _codon in synonymous_codons[codon])))

sum_codons={}

for codon in table5:

sum_codons[codon]=[counts[codon],result[codon]]

return sum_codons

def get_seq3(remainder, sequence, header):

r = remainder

line_new = sequence[:-r]

stop_codon = sequence[-r:]

len_seq2 = len(line_new)

remainder2 = len_seq2 % 3

print(header + ' ' + "incomplete stop codon" + ':' + stop_codon + ';' + str(r) + '>>' + str(remainder2))

return line_new

def read_fas_out_mul3(input):

name = input.split(".")[0]

ofile = name + '_mul3.fas'

outputfile = str(ofile)

# print(outputfile)

out_fas = open(outputfile, 'a')

with open(input) as input_f:

#fasta = {}

for line in input_f:

line = line.strip()

if line[0] == '>':

id = line[1:]

id2 = id.split(';')

header = id2[1]

else:

sequencelist=[]

sequencelist.append(line)

sequence = line

len_seq = len(sequence)

#enter the sequence name

out_fas.write('>' + header + '\n')

#calculates whether the sequence is a multiple of 3

remainder = len_seq % 3

if remainder == 0:

seq = sequence

else:

seq = get_seq3(remainder, sequence, header)

out_fas.write(seq + '\n')

input_f.close()

out_fas.close()

return outputfile

##plot_RSCU_bar

def make_stacked_plot(df_color):

# color

color = {1: '#FFFFAA', 1.5: '#FF9D6F', 2: '#0080FF', 2.5: '#FF2D2D', 3: '#E800E8', 3.5: '#00DB00', 4: '#8600FF',

4.5: '#FF5809', 5: '#EAC100', 5.5: '#0072E3', 6: '#FF0080'}

# canvas

plt.figure(figsize=(15, 10))

# quadrant 1

plt.subplot(211)

# rscu table perspective

dfrscu = df_color.pivot_table(index="AA", columns='color', values='RSCU', aggfunc=sum, fill_value=0)

#sets the bottom value of the stack plot and creates a list of color values

margin_bottom = np.zeros(len(df_color['AA'].drop_duplicates()))

heights_list = df_color['color'].drop_duplicates()

# stacking

for num, h in enumerate(heights_list):

values = list(dfrscu[h])

plt.bar(x=dfrscu.index, height=dfrscu[h].fillna(0), bottom=margin_bottom, facecolor=color[h], label=h, alpha=1)

margin_bottom += values

# Y-axis heading

plt.ylabel("RSCU")

# set the xy axis range

plt.ylim((-0.5, max(margin_bottom) + 0.5))

plt.xlim((-1, len(dfrscu.index)))

# quadrant 2

plt.style.use('ggplot')

ax2 = plt.subplot(212)

# set the 2nd quadrant background blank

ax2.patch.set_facecolor('none')

# codnon table perspective

dfcodon = df_color.pivot_table(index="AA", columns='color', values='codon', aggfunc=sum, fill_value='')

# xy location with codon content

xs = list(range(0, len(dfcodon.index.values)))

aas = dfcodon.index.values

ys = dfcodon.columns.values

# set the xy axis range and remove the xy axis

plt.ylim((min(dfcodon.columns.values) - 0.5, max(dfcodon.columns.values)))

plt.xlim((-1, len(dfcodon.index)))

plt.xticks([])

plt.yticks([])

# codon mapping diagram

for x in xs:

for y in ys:

ax2.text(x, y, dfcodon.iloc[x][y], va="center", ha="center",

bbox=dict(boxstyle="round", facecolor=color[y], alpha=1))

# save picture

plt.savefig(fname="RSCU.svg", format="svg")

plt.savefig(fname="RSCU.png", format="png", dpi=300)

plt.show()

def RSCU_process(input_fasta):

mul3_seq = read_fas_out_mul3(input_fasta)

abb = (input_fasta).split(".")[0]

seqs = [rec.seq for rec in SeqIO.parse(mul3_seq, 'fasta')]

rscu = RSCU(seqs, table5)

# statistical result

df_rscu_sum = pd.DataFrame(columns=['AA', 'codon', 'count_num', 'rscu_val'])

for codon in rscu.keys():

info_list = [table5[codon], codon, rscu[codon][0], round(rscu[codon][1], 3)]

df_rscu_sum.loc[codon] = info_list

df_rscu_sum.to_excel('{}_sum.xlsx'.format(abb))

# draw a picture

df_rscu_color = pd.DataFrame(columns=['AA', 'codon', 'RSCU', 'color'])

color = {}

for codon in rscu.keys():

if table5[codon] not in color:

color[table5[codon]] = 6

else:

color[table5[codon]] -= 0.5

info_list = [table5[codon], codon, round(rscu[codon][1], 3), color[table5[codon]]]

df_rscu_color.loc[codon] = info_list

df_rscu_color['codon'] = df_rscu_color['codon'].str.replace('T', 'U')

make_stacked_plot(df_rscu_color)

df_rscu_color.to_excel('{}_color.xlsx'.format(abb))

##base_count_ratio

def count_seq(sequence):

codon1 = []

codon2 = []

codon3 = []

total = len(sequence)

sequence = [sequence[i:i + 3].upper() for i in range(0, len(sequence), 3)]

for codon in sequence:

codon1.append(codon[0])

try:

codon2.append(codon[1])

except:

continue

try:

codon3.append(codon[2])

except:

continue

count_codon1=Counter(codon1)

count_codon2=Counter(codon2)

count_codon3=Counter(codon3)

seqA=count_codon1['A']+count_codon2['A']+count_codon3['A']

seqT=count_codon1['T']+count_codon2['T']+count_codon3['T']

seqC=count_codon1['C']+count_codon2['C']+count_codon3['C']

seqG=count_codon1['G']+count_codon2['G']+count_codon3['G']

#total=seqA+seqT+seqC+seqG

#dict_seq={'A':seqA, 'T':seqT, 'C':seqC, 'G':seqG}

'''

dict_c1=count_codon1

dict_c2=count_codon2

dict_c3=count_codon3

'''

#codon1的ATCG

cod1A=count_codon1['A']

cod1T=count_codon1['T']

cod1C=count_codon1['C']

cod1G=count_codon1['G']

post_1 = len(codon1)

#post_1 = cod1A + cod1T + cod1C + cod1G

#dict_c1 = {'A': codon1A, 'T': codon1T, 'C': codon1C, 'G': codon1G}

#codon2的ATCG

cod2A=count_codon2['A']

cod2T=count_codon2['T']

cod2C=count_codon2['C']

cod2G=count_codon2['G']

post_2 = len(codon2)

#post_2 = cod2A + cod2T + cod2C + cod2G

#dict_c2 = {'A': codon2A, 'T': codon2T, 'C': codon2C, 'G': codon2G}

#codon3的ATCG

cod3A=count_codon3['A']

cod3T=count_codon3['T']

cod3C=count_codon3['C']

cod3G=count_codon3['G']

post_3 = len(codon3)

#post_3 = cod3A + cod3T + cod3C + cod3G

#dict_c3 = {'A': codon3A, 'T': codon3T, 'C': codon3C, 'G': codon3G}

#[dict_seq,dict_c1,dict_c2,dict_c3]

return [seqA,seqT,seqC,seqG,total,

cod1A,cod1T,cod1C,cod1G,post_1,

cod2A,cod2T,cod2C,cod2G,post_2,

cod3A,cod3T,cod3C,cod3G,post_3]

def get_per_tab(input_table):

pertable=input_table.copy()

#print(input_table)

# pertable.insert(1,column='A%',value=(sum_table['A']/sum_table['Total'])*100)

pertable["A%"] = (pertable['A'] / pertable['Total']) * 100

pertable["T%"] = (pertable['T'] / pertable['Total']) * 100

pertable["C%"] = (pertable['C'] / pertable['Total']) * 100

pertable["G%"] = (pertable['G'] / pertable['Total']) * 100

pertable["AT%"] = ((pertable['A']+pertable['T'])/ pertable['Total']) * 100

# 'A-1', 'T-1', 'C-1', 'G-1', 'Post-1',

pertable["A-1%"] = (pertable['A-1'] / pertable['Post-1']) * 100

pertable["T-1%"] = (pertable['T-1'] / pertable['Post-1']) * 100

pertable["C-1%"] = (pertable['C-1'] / pertable['Post-1']) * 100

pertable["G-1%"] = (pertable['G-1'] / pertable['Post-1']) * 100

pertable["AT-1%"] = ((pertable['A-1'] + pertable['T-1']) / pertable['Post-1']) * 100

pertable["A-2%"] = (pertable['A-2'] / pertable['Post-2']) * 100

pertable["T-2%"] = (pertable['T-2'] / pertable['Post-2']) * 100

pertable["C-2%"] = (pertable['C-2'] / pertable['Post-2']) * 100

pertable["G-2%"] = (pertable['G-2'] / pertable['Post-2']) * 100

pertable["AT-2%"] = ((pertable['A-2'] + pertable['T-2']) / pertable['Post-2']) * 100

pertable["A-3%"] = (pertable['A-3'] / pertable['Post-3']) * 100

pertable["T-3%"] = (pertable['T-3'] / pertable['Post-3']) * 100

pertable["C-3%"] = (pertable['C-3'] / pertable['Post-3']) * 100

pertable["G-3%"] = (pertable['G-3'] / pertable['Post-3']) * 100

pertable["AT-3%"] = ((pertable['A-3'] + pertable['T-3']) / pertable['Post-3']) * 100

return pertable

def get_sum_table(input_table):

sumtable=input_table.copy()

forward_strand_table = sumtable[(sumtable['strand'] == '(+)')]

reverse_strand_table = sumtable[(sumtable['strand']=='(-)')]

sumtable.loc['totoal_sum'] = sumtable[['A', 'T', 'C', 'G', 'Total', 'A-1', 'T-1', 'C-1', 'G-1',

'Post-1', 'A-2', 'T-2', 'C-2', 'G-2', 'Post-2', 'A-3', 'T-3', 'C-3',

'G-3', 'Post-3']].apply(lambda x: x.sum())

#+

if len(sumtable[(sumtable['strand']=='(+)')]) > 0 :

sumtable.loc['forward_strand'] = forward_strand_table[['A', 'T', 'C', 'G', 'Total', 'A-1', 'T-1', 'C-1', 'G-1',

'Post-1', 'A-2', 'T-2', 'C-2', 'G-2', 'Post-2', 'A-3', 'T-3', 'C-3',

'G-3', 'Post-3']].apply(lambda x: x.sum()).copy()

else:

print("no (+) strand gene")

#-

if len(sumtable[(sumtable['strand']=='(-)')]) > 0 :

sumtable.loc['reverse_strand'] = reverse_strand_table[['A', 'T', 'C', 'G', 'Total', 'A-1', 'T-1', 'C-1', 'G-1',

'Post-1', 'A-2', 'T-2', 'C-2', 'G-2', 'Post-2', 'A-3', 'T-3', 'C-3',

'G-3', 'Post-3']].apply(lambda x: x.sum()).copy()

else:

print("no (-) strand gene")

final_table=get_per_tab(sumtable)

final_table[['A%', 'T%', 'C%', 'G%', 'AT%', 'A-1%', 'T-1%', 'C-1%','G-1%', 'AT-1%', 'A-2%', 'T-2%', 'C-2%',

'G-2%', 'AT-2%', 'A-3%','T-3%', 'C-3%', 'G-3%', 'AT-3%']]=final_table[['A%', 'T%', 'C%', 'G%',

'AT%', 'A-1%', 'T-1%', 'C-1%','G-1%', 'AT-1%', 'A-2%', 'T-2%', 'C-2%', 'G-2%', 'AT-2%', 'A-3%',

'T-3%', 'C-3%', 'G-3%', 'AT-3%']].astype('float').round(2)

final_table[['A', 'T', 'C', 'G', 'Total', 'A-1', 'T-1', 'C-1', 'G-1','Post-1', 'A-2', 'T-2', 'C-2',

'G-2', 'Post-2', 'A-3', 'T-3', 'C-3','G-3', 'Post-3']] = final_table[['A', 'T', 'C',

'G', 'Total', 'A-1', 'T-1', 'C-1', 'G-1','Post-1', 'A-2', 'T-2', 'C-2', 'G-2', 'Post-2',

'A-3', 'T-3', 'C-3','G-3', 'Post-3']].astype('int')

return final_table

def get_seqs_count(input_fasta):

base_count_dftmp=DataFrame(

columns=['strand',

'A', 'T', 'C', 'G', 'Total',

'A-1', 'T-1', 'C-1', 'G-1', 'Post-1',

'A-2', 'T-2', 'C-2', 'G-2', 'Post-2',

'A-3', 'T-3', 'C-3', 'G-3', 'Post-3'])

#count—seq

for rec in SeqIO.parse(input_fasta, 'fasta'):

sequence=rec.seq

id=(rec.id).split(';')[1]

#print(id)

strand=(rec.id).split(']')[1]

base_count_list=count_seq(sequence)

base_count_list.insert(0, strand)

base_count_dftmp.loc[id] = base_count_list

#sum_table.to_excel("{0}_{1}__{2}.xlsx".format(prefix, line, data))

count_table=base_count_dftmp.sort_values(by=['strand'], ascending=False)

final_sum_table=get_sum_table(count_table)

abb = str(input_fasta)

final_sum_table.to_excel('{}_base_count.xlsx'.format(abb))

if __name__ == "__main__":

parser = argparse.ArgumentParser()

parser.add_argument('--input', '-i',

type=str,

help='input file in fasta format')

args = parser.parse_args()

RSCU_process(args.input)

get_seqs_count(args.input)

print("Be careful! If the total length is not equal to the sum of ATCG,"

" ambiguous bases exist in the sequence , eg. Y K M ")
